# A new typology for understanding context: qualitative exploration of the model for understanding success in quality (MUSIQ)

**DOI:** 10.1186/s12913-018-3348-7

**Published:** 2018-07-25

**Authors:** Julie E. Reed, Heather C. Kaplan, Sharif A. Ismail

**Affiliations:** 1 National Institute of Health Research (NIHR) Collaboration for Leadership in Applied Health Research and Care (CLAHRC) Northwest London, Imperial College London, Chelsea and Westminster Hospital, 369 Fulham Road, London, SW10 9NH UK; 2 Perinatal Institute and James M. Anderson Center for Health Systems Excellence, Cincinnati Children’s Hospital, Cincinnati, OH USA; 3 Department of Primary Care and Public Health, Imperial College London, Reynolds Building, St Dunstan’s Road, London, W6 8RP UK

**Keywords:** Quality improvement, Context, Implementation, Qualitative research, Complexity

## Abstract

**Background:**

The importance of contextual factors in influencing quality improvement and implementation (QI&I) initiatives is broadly acknowledged. Existing treatments of context have primarily viewed it as static and distinct from interventions themselves. The objective of this study was to advance understanding of the complex and dynamic interaction between context, intervention, and implementation strategies. Using the Model for Understanding Success in Quality (MUSIQ), we aimed to better understand the roles of, and inter-relationships between, contextual factors within QI&I initiatives.

**Methods:**

Secondary analysis was performed on qualitative data collected as part of two studies: (1) an evaluation of a state-wide obstetrical quality improvement (QI) initiative, and (2) a study of the use of Plan-Do-Study-Act cycle method in QI projects. Electronic coding databases from each study were reviewed jointly. Data analysis was initiated deductively using MUSIQ as a template. Codes were added in an inductive manner.

**Results:**

All original factors in MUSIQ were observed to be important in the QI initiatives studied and new factors were identified. Three distinct types of context were identified; the setting(s) of care in which QI&I takes place (Type 1); the context of the team conducting a specific project (Type 2); and the wider context supporting general QI&I (Type 3). The picture of context emerging from this study is a dynamic one with multiple, closely-linked factors operating at different levels in a system that is constantly changing in response to QI&I initiatives. To capture this complexity, a revised model (MUSIQ v2.0) was created positioning use of structured QI&I approaches as the focal point and demonstrating how context influenced effective use of these approaches, and in turn, how these approaches supported teams in navigating context by adapting interventions to fit local settings.

**Conclusions:**

MUSIQ is a useful tool to explore the roles of, and inter-relationships between, contextual factors within QI&I initiatives. The revised model may help address some existing controversies about how context influences QI&I success and help ensure that future research efforts consider context not as static background, but as a complex system that is constantly changing, tightly-linked, and governed by feedback loops.

**Electronic supplementary material:**

The online version of this article (10.1186/s12913-018-3348-7) contains supplementary material, which is available to authorized users.

## Background

The history of quality improvement and implementation (QI&I) in healthcare is replete with examples of initiatives that show either considerable variability in impact across settings, or an inability to replicate previous successes when applied in new arenas [1]. The context in which improvement initiatives are implemented is frequently cited as an explanation for this variability [2, 3], but we have limited understanding of what this means in practice. In part, this is because much research continues to focus on technical aspects of interventions (what works) and the process of implementation (how it works). In addition, there are significant boundary challenges in defining context. While context is typically recognised as everything that surrounds an intervention or improvement effort [4], in reality, the boundaries between an intervention, the environment in which it is delivered, and its effectiveness are blurred [5]. Finally, despite the growing body of evidence demonstrating that the context in which interventions are introduced influences their successful implementation, those examining the role of contextual factors in QI&I initiatives conventionally treat contextual factors as static, background elements, with little reference to the complexity of the environments into which interventions are introduced.

A range of heuristics have been proposed to understand the role of context in implementation and improvement efforts including the Promoting Action on Research in Health Services framework, [6] the Consolidated Framework for Implementation Research, [7] Receptive Contexts for Change, [8] Organisational Readiness for Change, [9] Emotional Dynamics, [10] Organising for Quality, [11] the Knowledge to Action Cycle, [12] and the National Health Service (NHS) Sustainability Model [13] among others. The emergence of a large number of models attempting to explain similar phenomenon may be a result of the difficulty in codifying a concept that is inherently complex. Researchers are challenged by trying to draw clear boundaries around systems which may be subject to a variety of social contextual and organisational factors (among others) at different times [14]. Secondly, researchers disagree on the extent to which context is considered concrete and measurable versus something that is perceived and socially constructed [15, 16]. Thirdly, there is confusion about the extent to which context can be described by lists of important factors, versus the extent to which such factors are dynamic and interact with each other, the intervention and implementation process [15, 17–19]. Perhaps one of the most important disputes is the extent to which different aspects of context are navigable or modifiable [20–22].

More recent work applying complexity science to better understand the impact of context in QI&I interventions has the potential to provide the framing needed to better align the field [23]. Recent work by Hawe and colleagues, [24, 25] as well as work by May, [14] advances a dynamic understanding of the relationships between an intervention – modelled as a series of events – and the context in which it is implemented. This work – which focuses on context as a complex adaptive system – has the potential to transform our understanding of how and why interventions work in some settings and not others [14]. Actors in a health system are understood to respond individually and collectively to changes in their environment in a dynamic way over time, and in ways that may eventually result in new behaviours at a system-level (self-organising behaviour) [25, 26].

Although there is growing interest in the potential of insights from complex systems theory to inform our understanding of context in QI&I, real-world applications of systems thinking to this problem have been few and far between. This study employs the Model for Understanding Success in Quality (MUSIQ) [27] to help understand context. MUSIQ was originally derived from a systematic review of quantitative evidence [28] and expert consensus [27]. It has subsequently been tested in an exploratory quantitative analysis of 74 Quality Improvement (QI) projects [29]. MUSIQ identifies 25 contextual factors across multiple nested levels of the healthcare system. Importantly, it also suggests key relationships among these factors. MUSIQ is distinguished from the other models currently guiding this area of research (those models listed above) by its attention both to generating a taxonomy of the contextual factors affected QI&I initiatives, and in explicitly recognising the dynamic and time-contingent nature of interactions between the contextual factors, and between the context and the interventions when implemented in a complex system.

The objective of this study is to advance the understanding of the complex and dynamic interaction between context, intervention, and implementation strategies. Using the Model for Understanding Success in Quality (MUSIQ), we aimed to better understand the roles of, and inter-relationships between, contextual factors within improvement and implementation initiatives using qualitative data from a series of QI&I efforts and to present an evolution of the MUSIQ model in light of these findings.

## Methods

This study employed secondary data analysis of qualitative research undertaken as part of two independent studies. Both studies used MUSIQ as a model to guide study design and interview constructs. Study A was a process evaluation of a collaborative QI programme. Study B was a qualitative study on the use of plan-do-study-act cycles (PDSA) in QI projects and the influence of context on effective use of PDSA cycles.

### Methods for study a [30]

Data were collected as part of a process evaluation of an Ohio Perinatal Quality Collaborative (OPQC) QI initiative to improve birth registry accuracy and reduce elective deliveries before 39 weeks in Ohio maternity hospitals [institutional review board approved 2013-0705]. Seventy-two hospitals were divided into three balanced groups that participated in monthly 1:1 coaching webinars with an OPQC QI facilitator for the first 3 months, a single face-to-face learning session designed to build community, and monthly group webinars to share ideas and track progress. As part of the process evaluation, the QI operational team leader (QI team leader) and obstetrician lead from a sample of hospitals were invited to participate in telephone interviews conducted within 1-month of project completion. Open-ended questions and follow-up probes were used to understand: how and why the hospital chose to participate in the OPQC project; changes made as part of the OPQC project; aspects of local context that were barriers or facilitators of the project; and ways in which the hospital supports, or does not support QI. Data analysis was initiated with a deductive approach, utilizing the question guide as an initial template for the coding framework, and then proceeded in an inductive manner [31, 32]. Codes and supporting quotes from transcripts were recorded in an electronic database (Microsoft Excel 2007).

### Methods for study B [33, 34]

Data were collected as part of an international study to explore the use of PDSA cycles in experienced QI organisations. [research ethics committee reference: 13/WM/0436] Two hospitals participated (located in Scotland and USA) and four active QI projects were identified from each site. An ethnographic approach was utilised including interviews, observations and document analysis. Multiple staff were interviewed from each project team, ranging from supporting QI staff to organisational senior leaders. Open-ended questions and follow-up prompts were used to understand the use of QI methods including PDSA cycles, the facilitators and barriers to their use and how this was affected by contextual factors. Data analysis was initiated with a deductive approach, utilizing the principles of PDSA conduct [35] and MUSIQ as theoretical models, and then proceeded in an inductive manner [36, 37]. Codes and supporting quotes from transcripts were recorded in an electronic database (Nvivo 10).

### Secondary analysis

In total over 50 h of interviews were coded from 59 staff (13 from Study A; 46 from Study B) from 14 project teams (8 from Study A; 6 from Study B) in 10 different organisations. (8 from study A; 2 from Study B). In addition, 16 coaching calls and 4 group webinars from Study A documented PDSA cycles in Study B were coded. Thematic analysis was conducted against items in the original MUSIQ model. In addition to deductively coding factors already present in the MUSIQ model, authors also used inductive coding to identify new factors and understand relationships between factors and successful QI&I. Coding was conducted independently with two coders (HK and JR) regularly coming together to review coding and discuss and agree on new coding items and definitions. Through a number of iterations, a final coding structure was agreed upon. At this point coders reviewed each other’s coding in full to ensure that definitions were understood. Any disagreements were resolved by consensus.

## Results

All original factors in MUSIQ were observed in both qualitative studies. New factors, not previously included in MUSIQ, were also identified. Contextual influences at all levels (microsystem, QI&I team, infrastructure, organization, and external environment) were noted and there were many examples of important relationships between contextual factors as hypothesized in MUSIQ [38]. We present in detail the key contextual factors discussed by participants in these studies, evidence supporting the complexity of the relationships between all factors that were observed, and an overview of three different types of context that we identified in our analysis.

### Key contextual factors

All factors previously identified in MUSIQ were identified in our qualitative exploration. Analysis contributed to alterations to original contextual factors for three reasons: broadening the definition of original factors (*n* = 2); increasing specificity of original factors (*n* = 5) to clarify whether they related to project specific or general factors; and the introduction of new factors not previously included in MUSIQ (*n* = 6). The terminology of all factors was modified from QI to QI&I to make explicit the application of the model to both quality improvement and implementation initiatives. A total of 36 contextual factors were described in the qualitative data examined from these two distinct studies. A full list of contextual factors identified with associated definitions is provided in Additional file 1.

Two of the existing MUSIQ factors were modified to broaden their definitions. *Senior Project Sponsor* was renamed *Organisational Leadership (project specific)* to reflect the fact that organisational leadership for a specific project may be required from multiple individuals rather than an individual project sponsor. *Physician involvement* was renamed to *Physician and Clinician Involvement* to recognise the role of other healthcare professionals, including nurses and allied health professionals, in supporting certain QI&I efforts.

Analysis revealed that five of the original MUSIQ factors could be clarified by further specifying whether the factor had a project specific or general context influence. For example, in the original MUSIQ model, the factor *External Motivators* was defined as the motivation to participate in a specific project. Our analysis revealed that as well as identifying external factors that influence motivation for specific projects (e.g. a national target for a particular care pathway), external motivators could also influence the wider organisation (e.g. government incentives for adopting an organisation wide QI&I approach). We therefore redefined *External Motivators* as two factors *External Motivators for QI&I* and *External Motivators (project specific*). This distinction was considered important as the sources of motivation for broader QI&I versus specific projects were often different, and influenced success through different mechanisms. Four other factors from the original MUSIQ model were also amended to reflect their different relationship to QI&I generally or to specific projects. *Payment structure (project specific)* and *QI&I payment structure* were more clearly defined to delineate the difference between payment arrangements that support participation in a specific project versus compensation structures that support broader engagement in QI&I. *Data infrastructure* was amended to reflect that infrastructure to support data can relate to the degree in which data is available and accessible to support a specific improvement project (*Data Availability (project specific)*) and the extent to which systems exists to collect, manage, and facilitate the use of data needed to support improvement more broadly *(Data infrastructure for QI&I)*. For example:

- **Data availability (project specific)**: *“[the collaborative Quality Improvement Consultant] helped us to get that additional data to help us identify our issues …she was able to help us get patient level information which showed the patients that were being considered,...elective deliveries.”* (P2 (QI team leader), Org 1, Study A)
- **Data infrastructure for QI&I: “** *[the performance improvement department] help extract the data [for our QI dashboard], collect the data, put it in a format that we can review …”* (P13 (QI team leader), Org 8, Study A)

Similarly, *Microsystem Culture* was added as a counterpart to the original factor *Microsystem QI&I Culture* to reflect the wider beliefs, values and relationships within a microsystem that affect the ability to make changes in a specific project as opposed to a microsystem’s more general commitment to improve. *Microsystem Leadership (project specific),* defined as the leadership’s involvement, support and facilitation of a specific project, was added as a counterpart to the original factor *Microsystem QI&I Leadership* which relates to the ways in which microsystem leaders support QI&I in general as exemplified by:

- **Microsystem leadership (project specific):** *“…our chair has been very clear to our providers and…actually called them on it [delivering before 39 weeks without an indication] … he’s actually gone through and talked to providers individually about it if they were not following ...”* (P13 (QI team leader), Org 8, Study A)
- **Microsystem QI&I Leadership:** *“Our first meeting with [a department physician leader], was like...run charts and PDSA cycles...oh. But now he’s like a convert... it’s like his department and his area, and he’s really taken ownership of it.”* (P43 (QI manager), Org 10, Study B)

In addition to the modifications made to more clearly define the existing MUSIQ factors, six entirely new factors were identified based on the qualitative data examined in this study. The new factors along with representative quotations illustrating the importance of these factors are detailed in Table 1.

**Table 1:** New context factors

New Factor	Description	Representative Quotation(s)
External	The extent to which the team or organisation values and acquires QI&I knowledge from external sources (e.g. publications, other QI&I organisations)	*“… then learning from others as well... our institution is always looking at what others are doing, how can we learn from others and, you know, … no one person is doing any of this... we have a whole team looking at evidence and measures, and they’re learning from people.”* (P22 (senior QI manager) Org 9 Study B)
External Knowledge (general QI&I)		
External	The extent to which the team or organisation values and acquires project related knowledge from external sources (e.g. guidelines, other project teams, other hospitals)	*“… with those, the forms that we created and we just kind of modelled that after some of the forms that we saw … we just kind of took things that other institutions were doing to create our own form”* (P6 (QI team leader),Org 4, Study A)
External Knowledge (project specific)		
Infrastructure	The extent to which an organisation has a system for managing multiple QI&I projects including processes for selecting projects and appropriate QI&I methods to use	*“I think we struggle a lot with that balance, and so we… do hold back and really are careful when we decide we do want to test something, because we have multiple improvement projects going on in the same area. So, we need to know which ones overlap…in which ways, how will they then affect the other tests and ultimately the patient, and so we… We have to make a very conscious effort to limit some of our testing.”* (P23 (QI specialist) Org 9 Study B)
QI&I Portfolio Management		
		*“…we basically are following three different sets of guidelines out there [Medicare, state collaborative, Joint Commission]… And it makes it even very hard for our quality management department because we have to remember which one we are reporting for and which set of guidelines we are using for our numerator and denominator definitions.”* (P2 (QI team leader),Org 1, Study A)
Infrastructure	The availability, expertise and experience of staff with specialist or high levels of QI&I knowledge and skills	*“We did have very specific and trained teams…who had one of three roles – they were either a process person, to help with process improvement, or they were an [electronic health record (EHR)] analyst, to help with the [EHR] changes, or they were a data requirements person, to help with writing and programming all of the measures.”* (P22 (senior QI manager), Org 9, Study B)
Specialist QI&I Staff		
		*“Definitely, [Quality Department staff]… The fact that she was willing to be part of the team, she did a lot of work …pulling everything together.”* (P9 (QI team leader), Org 6, Study A)
Microsystem	Microsystem staff’s collective potential for delivering care and executing QI&I projects	*“…Simultaneous changes happening at once. It’s really… It’s very challenging to keep track of all that when you’re working and that is happening as well as taking care of large volumes of patients...So it’s not just all of your responsibilities and duties of your day, but then you have to keep in mind of all these other things that you need to do for that day as well….I think sometimes it just gets so incredibly busy that it either gets away from you or you just can’t fit another thing on your plate. I mean, you know, and that’s the reality of it.”* (P25(nurse), Org 9, Study B)
Microsystem Capacity (project specific)		
		*“… The process needs to be something that they can fit into what they do every day. And it can’t be in addition to. You know, if all of a sudden you’re going to say to somebody, I know you’re really, really busy, but I want you to do something else as well… and we do that a lot. So if you could just do this as well. And then it doesn’t happen…*” (P45 (service manager), Org 10, Study B)
QI&I Team	Contribution of patients, carers and members of the public to the QI&I team efforts	*“And then engaging family partners, that’s been one of our priorities…to have a more intentionality around identifying families who wanted to be, or adult patients, who wanted to be partners in the work, and they’re volunteers, but we do have at least two; some teams have more families that are partners.”* (P22(senior QI manager), Org 9, Study B)
Patient Engagement & Involvement		

### Relationship between context factors

The original MUSIQ model proposed a number of relationships between factors to support a deeper understanding of the mechanisms of action by which context influences QI&I success. In exploring in-depth qualitative accounts of QI&I efforts, the authors were able to identify examples of these relationships. In many cases, both positive and negative examples were identified, for example describing the benefit of having organisational *Data Infrastructure for QI&I* on *QI&I team Decision Making* as well as the challenges when such infrastructure didn’t exist. In addition, we found examples of two-way relationships, for example *Microsystem QI&I Leadership* was able to influence *Organisational QI&I Leadership* as well as vice versa.

**Fig. 1:**
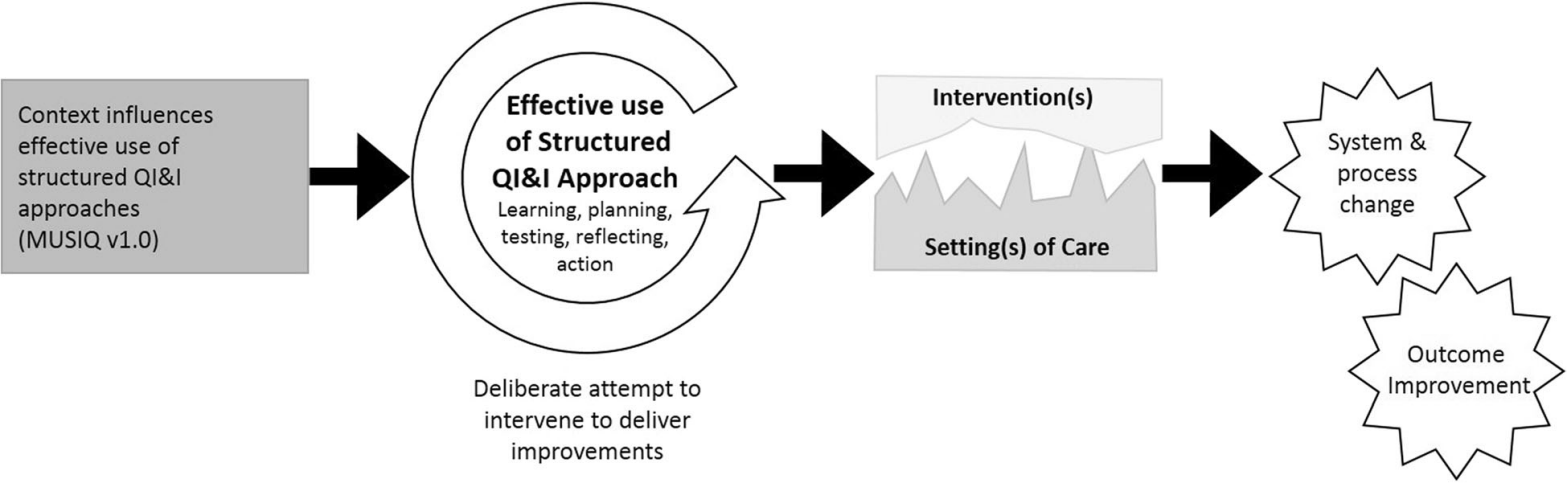
A schematic showing the effective use of quality improvement approaches as a focal point for our analysis in understanding how context influences the effective use of structured QI&I approaches, and how in turn these approaches support navigation and negotiation of change within local contexts in order to achieve system, process, and outcome improvement

Key relationships that existed in the data related to how improvement is mediated through use of systematic, structured QI&I approaches. The use of structured QI&I approaches influence, and are influenced by, contextual factors in a non-linear way (Fig. 1). In the original MUSIQ model success was depicted as the implementation of system and process changes with resulting outcome improvement. The use of a structured QI&I approach to implement system and process changes was implied, but not explicitly defined. Qualitative data from this study demonstrated the importance of having a structured approach to support learning, planning, testing, reflection and action to introduce an effective change through an iterative process that adequately accounts for contextual factors. Effective use of a structured approach was observed to support teams in understanding and navigating the context or setting(s) of care in which evidence or other intervention(s) are introduced, and negotiating the changes that need to take place. On the other hand, the context of the team conducting a specific project and the wider context supporting general QI&I were observed to influence how effectively a team was able to utilise a structured approach to iteratively learn, test changes at a small scale and then implement broadly. Examples of these relationships are provided in the following section.

#### The local care setting is navigated by the use of structured QI&I approaches

Use of a structured QI&I approach enables teams to adapt interventions to the local setting or context. This ensures that interventions fit with practices and processes of care and allows QI teams to address related problems or issues that prevent the intervention(s) from being implemented and from the improvement goal being reached. For example, adapting interventions so that they are understandable for staff based on a range of knowledge levels is an illustration of an important adjustment based on local setting of care:

*“[We developed] an initial assessment form so that it helps guide their treatment for that patient that day …[but] they [the staff] didn’t know what those initials were…so that’s why we had to …go back and put the instructions and spell everything out to make it a little more understandable.”* (PDSA Documentation, Org 9, Study B)

Similarly, using a systems’ lens to view change, as encouraged in QI methodology, can help teams identify and institute changes to related practices or processes that needed to be made to support implementation of the target intervention, by recognising the degree of connectivity between agents and processes in the system. Our data suggest this was particularly important when implementation of the target intervention was dependent on work that happens in other parts of the mesosystem.

*“If you have an induction less than 39 weeks, you have to specifically state why it’s occurring and then, our scheduling all goes through obstetrics now instead of both obstetrics and surgery for the C-sections.”* (P7 (OB lead), Org 5, Study A)

*“… at the same time our surgery department was doing changes with their scheduling…And so, the changes in our surgery department actually kind of coincided with what we need which was OK if one surgeon hasn’t scheduled anything and we have a C-section that needs to done, and it’s not their day. They still have an opportunity to do that, so that was kind of good that it went together.”* (P9 (QI team leader), Org 6, Study A)

However, the tightly-linked nature of intervention context was also emphasised by the frequency with which wider organisational issues linking to staff capacity and capability were identified in this study as barriers to introducing new ways of working. Organisational or external factors influencing staff availability could not always be influenced by the team, and as a result teams had to adapt their intervention and improvement attempts to work within available resources.

*“So we're going to have to go back and look at all the junior doctor rotas [schedules] because there's not enough of them [to deliver the intervention as planned]… So the system we've got in just now's not sustainable”* (P48 (QI specialist), Org10, Study B)

*“From an [emergency department] perspective one of the drivers in that is that there are no doctors, no-one’s in emergency medicine anymore,... we can’t recruit to emergency medicine so we’ve got to look at alternative models, other options. And one of them is a non-medical model of nurses and paramedics.”* (P41 (consultant), Org 10, Study B)

Effective use of structured QI&I approaches necessitates that teams engage with other frontline staff to surface locally held knowledge and opinions. Dialogue with frontline staff was seen to influence the changes made by the team because it enabled them to proactively identify and adapt to emerging barriers and opportunities communicated by frontline staff as they implemented process changes:

*“We brought our physicians in and discussed that [scheduled delivery] form and they basically [were] still pretty set in their ideas…And [with the conversation] they came to a better realization and understanding of…what their roles and responsibilities are in that....And they are going to provide additional education to the patient from the very beginning of the need to take this delivery…to at least the 39 weeks.”* (P2 (QI team leader), Org 1, Study A)

*“Because if I design it, it’s going to be worthless…. you’re going to have people saying, you know what, this doesn’t work because I can’t give them the urine cup because I have to sign them up for [the electronic health records]... So you find yourself negotiating…”* (P17 (senior QI manager), Org 9, Study B)

#### Project specific and general QI context influences how teams use structured QI&I approaches

##### Direct influence of project-specific context on the effective use of structured QI&I approaches

The context in which a specific project was being conducted had important influences on the ability of the team to use structured QI&I approaches—learning, reflection, planning, testing, and action—in order to support intervention, implementation and improvements in processes and outcomes of care. The ability of the team to learn and take actions through use of a structured approach is influenced both by the nature of the team (including their experience, skills and diversity), and by additional factors including the extent to which other people are committed and motivated to change, and wider meso- and macro- factors that influence levels of motivation and support. The specific project context therefore includes the social and psychological components of change. For example, a strong relationship between the team and microsystem staff facilitated productive testing and implementation of changes:

*“…the meeting that we had where we brought just the obstetricians and core members of this [QI] team, that we were able to share that information in a way that allowed them to ask questions and understand it better. Whereas, in the other meetings, they accepted the information and changed process to a certain extent but maybe never got quite as much of an understanding as they did from the meeting that we had when we were developing the form...That allowed open dialogue...”* (P2 (QI team leader), Org 1, Study A)

*“…I think the hardest part with the PDSAs in the group is communication… because the front-line staff, they’re the ones that are doing it…We discuss it at our [QI&I Team] meetings. We meet bi-weekly, and we’ll say, we’re going to test A, B and C. They’re not there…It’s your front-line folks that are doing it, so, really that communication, getting [the information out there]…”* (P17, (senior QI manager) Org 9, Study B)

One particular way that teams engage microsystem staff is through sharing data to motivate microsystem staff to engage with tests of change. Data availability for the specific project moderates this relationship:

*“Our folks respond much better if we can show them discrete data to indicate whether this did or did not work, … So, the degree in which you can process that data and turn it around fast and then use that to convince your audience.”* (P24 (QI specialist), Org 9, Study B)

*“… I showed him [obstetrical department chair] the data we had and what we did to change the birth certificate registry…he was concerned because that percentage was high, but he was at the OB [microsystem] meetings and he would reinforce what I had told them, what we had found.”* (P5 (QI team leader), Org 3, Study A)

Motivation among microsystem staff was a critical factor in successful improvement. If microsystem staff are not motivated, then the team will have difficulty implementing change and executing a cycle of learning to identify whether the changes they are interested in making result in improvement.

*“…the challenges are when you get to the point of having to convince a wider audience to do something that's different from what they do at the moment, the same issue of somehow getting them to come to the conclusion that it's a good idea to do it…if I tell them to do it and they don't think it's a good thing to do then…they don't do what you've asked them to do, [and the QI team] measure it and they demonstrate there has been no change because they [staff] have effectively done what they did before anyway...”*(P40 (medical director), Org 10, Study B)

Professional diversity within the team, particularly physician and clinician involvement, increased the motivation of microsystem staff to test changes.

*“I think the fact that we did multidisciplinary input on the tool really engaged people. And made it less foreign when we actually implemented it. And we did take a little time to get that done…but it was time well spent.”* (P1 (OB lead),Org 1, Study A)

*“I also feel that there’s an advantage in [my role as a nurse and QI&I team member]…because these are my peers that I’m trying to actively engage with, as opposed to a hierarchy or leadership where they feel the sense of, I have to do this because my manager’s telling me to. When I engage people in testing I’m, I’m just one of them…Nor do I think they hold back on me when they…feel like this is going to work or it’s not.”* (P26 (nurse), Org 9, Study B)

Support and motivation for a specific change effort may also be influenced by environmental factors such as external motivators for project participation (e.g. incentives, macro level competition) and project sponsorship. External project sponsorship can facilitate use of QI methods and other structured implementation approaches through building motivation, providing resources, and facilitating knowledge sharing around the project.

*“I felt a great support through the [QI Collaborative] webinars and it just increased my confidence level to just keep doing what I was doing and knowing that I was doing the right thing.”* (P8 (QI team leader), Org 5, Study A)

Particularly around efforts to reduce elective deliveries before 39 weeks (Study A), concerns about competition and potential for ties to pay for performance increased motivation:

*“...so they [physicians] really haven't squawked too much because I think in their profession, they recognized that this is… a national standard, [not our] Hospital telling you can't do delivery before 39 weeks...”* (P9 (QI team leader),Org 6, Study A)

*“Money…When you tell them that these things are probably not going to be covered if we don’t do it this way or not be paid for. Yes, kind of the crowbar.”* (P7 (OB lead), Org 5, Study A)

The influence of project specific context was also illustrated as the extent to which the organisation leaders provide support and resources to help the team overcome barriers and obstacles encountered during the specific project.

*“…the institution knew that we had a [birth registry] data issue…so I had support from them because I had some [nurse] hours wrapped up in clearing up data…”* (P11 (QI team leader), Org 7, Study A)

*“… I think my role [as member of the executive team] is a supportive one and to try and facilitate what it [the organisation] wants done…And when somebody says, why don't we do the following, stick my hand up at that point and say, yes, well, I can contribute to that by providing... I'll go and speak to so-and-so in a different department or find some secretarial support.”* (P40 (medical director), Org 10, Study B)

##### Indirect influence of wider general QI context on effective use of a structured QI&I approach

The ability of a team to effectively use structured QI&I approaches is influenced by the extent to which the wider organisational context is supportive of improvement and evidence implementation at micro, meso and macro levels. For example, *Organisation QI&I Culture* is defined as the values, beliefs, and norms of an organisation that shape the behaviours of staff in pursuing QI&I. A strong culture supportive of QI&I is reflected by staff that are willing and able to participate in QI&I approaches to test and implement changes.

*“I mean, if they know something is the right thing to do, they're going to do it, and I have to say that about our hospital that they're going to take the bull by the horns and take care of it when they see that it's important.”* (P5 (QI team leader),Org 3, Study A)

The impact of having a culture that supports QI&I more locally, at the microsystem level, impacts the receptivity and capability of the team and microsystem staff to engage in QI&I activities both broadly and as it relates to a specific project. These effects are mediated by microsystem motivation and QI&I capability:

*“... I remember when I first came [here], I was shocked that they had run charts and control charts on the wall and they knew what they were, they weren't just wallpaper”* (P19 (QI director), Org9, Study B)

*“I’m probably very, very lucky because I’ve a fab team … They come to me with an idea...on Monday this is what we’re going to try, the PDSA goes up, everybody’s aware of it, and so everybody’s on board.”* (P49 (nurse), Org 10, Study B)

Organisational strategies to develop the QI&I workforce impact the QI&I skill of the project team as well as the QI&I capability of microsystem staff which in turn impact the willingness and ability of the microsystem and QI&I team to engage in investigations and test of change:

*“…we just had a great [microsystem] director who really bought into QI, was very supportive, and they have a career ladder within nursing. So to move up, you have to… you take on more responsibility and leadership. So she used that as an opportunity to get people involved, so, you know, giving them opportunities to lead projects or be involved in projects…we worked really hard to build improvement capability in the unit, and so I think that was very helpful.”* (P16 (senior QI manager), Org 9, Study B)

Similarly, organisational strategies to invest and develop specialist QI&I support staff and place those individuals in supporting roles within the QI&I Team and microsystem, influence the effectiveness of using structured QI&I approaches. The additional skills and competencies provided by these individuals complemented the local QI&I team to support their effective use of structured QI&I approaches:

*“we have some teams where we’ll [QI&I specialists] function as, sort of, the QI experts and lead, sort of, coaching and lead the teams through change packages and things, and …teach them the process. We have other teams that have done this before…so it's less about, necessarily the coaching piece of it, and more of a okay are we evaluating properly, are we doing the process the way we want to…”*(P24 (QI specialist), Org9, Study B)

*“She [QI&I Department staff] was great looking at data because that’s kind of what she does with the quality stuff, so I would say…her support in that was really, really good.”* (P9 (QI team leader), Org 6, Study A)

The organisational approach to building a data infrastructure to support QI and broader evidence implementation, specifically providing resources for measurement and analysis, is critical in supporting the team in effective use of structured QI&I approaches:

*“the fact [is] that you do need a permanent resource…if you are really going to do this at the scale... So [our improvement work] would be nothing without [our data specialist] that sits out there producing the reams of data that’s on the wall there... even small things like trying to work with them [the organisation’s information analysts] to change the way they present audit data into measurement of time... you know, using run charts, statistical process control.... ”* (P46 (QI manager), Org 19, Study B)

As organisational interest in QI&I increases it can be difficult for existing data infrastructure systems to cope with the increased demand and thereby impacting data availability for specific projects:

*“Yes, the information services, to their credit, has taken a very open access policy to data. It may be technically difficult to get to things but there are channels for making requests and they really don’t obstruct it…the obstruction is just that there’s more requests than people can handle.”* (P21 (senior data analyst and manager), Org 9, Study B)

An organisation’s tactic for managing their portfolio of work impacts not only the organisation or microsystem’s capacity for conducting improvement work but also their motivation to engage in testing (if work is clearly aligned with microsystem and organisational goals)

*“…the overlapping and competing priorities of the teams created a lot of confusion for the frontline staff. And so the staff just [say] forget it…I'll do what I know I need to do, which is take care of the patient.”* (P26 (nurse), Org 9, Study B)

*“… so we do participate in the goal setting and the alignment with both the organisation and then what the divisional priorities are. We try to help figure that out so we’re not stressed in terms of the portfolio, we’re not overreaching every year”* (P24 (QI specialist), Org 9, Study B)

### Three types of context

A key observation that was identified from the data was that individuals, teams, and organizations were thinking about and addressing three different types of context: type 1 represents the context of the setting(s) of care where QI&I takes place (navigated and negotiated by the use of a structured QI&I approach); type 2 represents the project specific supporting context; and type 3 represents the wider QI&I context (contexts which influence the effective use of structured QI&I approaches). Each type of context is distinguished by its relationship to the use of structured QI&I approaches and its proximity to the specific intervention or improvement being made. The ways in which these three types of context emerged from the qualitative data are illustrated in Table 2 with an example from each study.

**Table 2:** Examples of Context Typology Described in Studies A and B

Context Type		Study A Example	Study B Example
		Study aim: Reduce elective delivery of babies before 39 weeks where no medical indication that early delivery is required	Study aim: Improve identification of high risk patients in Emergency Department to ensure treatments are delivered in a timely manner
Use of QI&I approach to support navigate and negotiate change	Type 1 Setting(s) of care in which a project takes place	Analysis of incidents of deliveries before 39 weeks gestation without a clear indication enabled a team to identify systemic operational issues related to ability to schedule deliveries on a weekend:	Developing an intervention to appropriately screen the local patient population required many iterative tests of change to ensure it was working effectively:
		*“We have a situation where we do not have operational support to do elective induction and delivery and C-sections on the weekends. And we are building a coalition to get the operational support around that...”* C2 (coaching call),Org 1, Study A	
			*“So there was a best practice alert [triggering at risk patients on arrival to Emergency Department], and we had it on in the background for two months, just testing it and nobody saw it…We would get data and... we would say these patients triggered, were they really the right patients to trigger? And then we continued to tweak the tool…”*P23 (QI specialist), Org 9, Study B
Influence of context on effective use of structured QI&I approach	Type 2 Project specific supporting context	Success in eliminating early elective deliveries necessitates buy-in from the obstetrical physicians; therefore, having the right QI team members to effectively engage with physicians was critical to test changes and learn what challenges were being faced:	Developing an effective screening tool required the QI team to obtain feedback from frontline staff on any problems experienced in practice. Staff knew their concerns would be listened to and this influenced their willingness to engage in test of changes:
		*“Physician support was really instrumental because I think if it was just coming from nursing or clerically from a secretary, there’s just no way, there would have been no buy-in.”* P6 (QI team leader), Org 4, Study A	*“Yes, I do think that we [staff] are listened to…Because any time I’ve sent an email, or I’ve said I feel like I’ve had trouble with, like if I’m screening somebody and there’s a problem, I get immediate email back that they’ve looked into it. Which I think is great.”* P25 (nurse), Org 9, Study B
	Type 3 General QI&I supporting context	The extent to which the organisation and microsystem had a general culture of providing standardised care influenced the ease of introducing the specific 39 week care standard:	In introducing the best practice alert screening system the QI team reflected how general QI capability among staff facilitated tests of change and how this had been influenced by organisational QI leadership:
		*“We really try hard to standardize everything… we really do try to structure everything and standardize it, make everything as fair as possible… just having a lot of policies and protocols in place, so that we’re always doing the same thing for one patient as we would the next...”* P6 (QI team leader), Org 4, Study A	
			*“I’m continually impressed by how much everybody actually knows [about QI methods]… So the barrier in terms of explaining things is not as high…*
			*…the fact that they are familiar with QI methods and run charts…it comes from the CEO on down …*. *it’s very common language.”* P24 (QI specialist), Org 9, Study B
		*“They don’t believe in it [standardization]. Absolutely. Yes, it is the Achilles heel.”* P7 (OB Lead), Org 5, Study A	

**Fig. 2:**
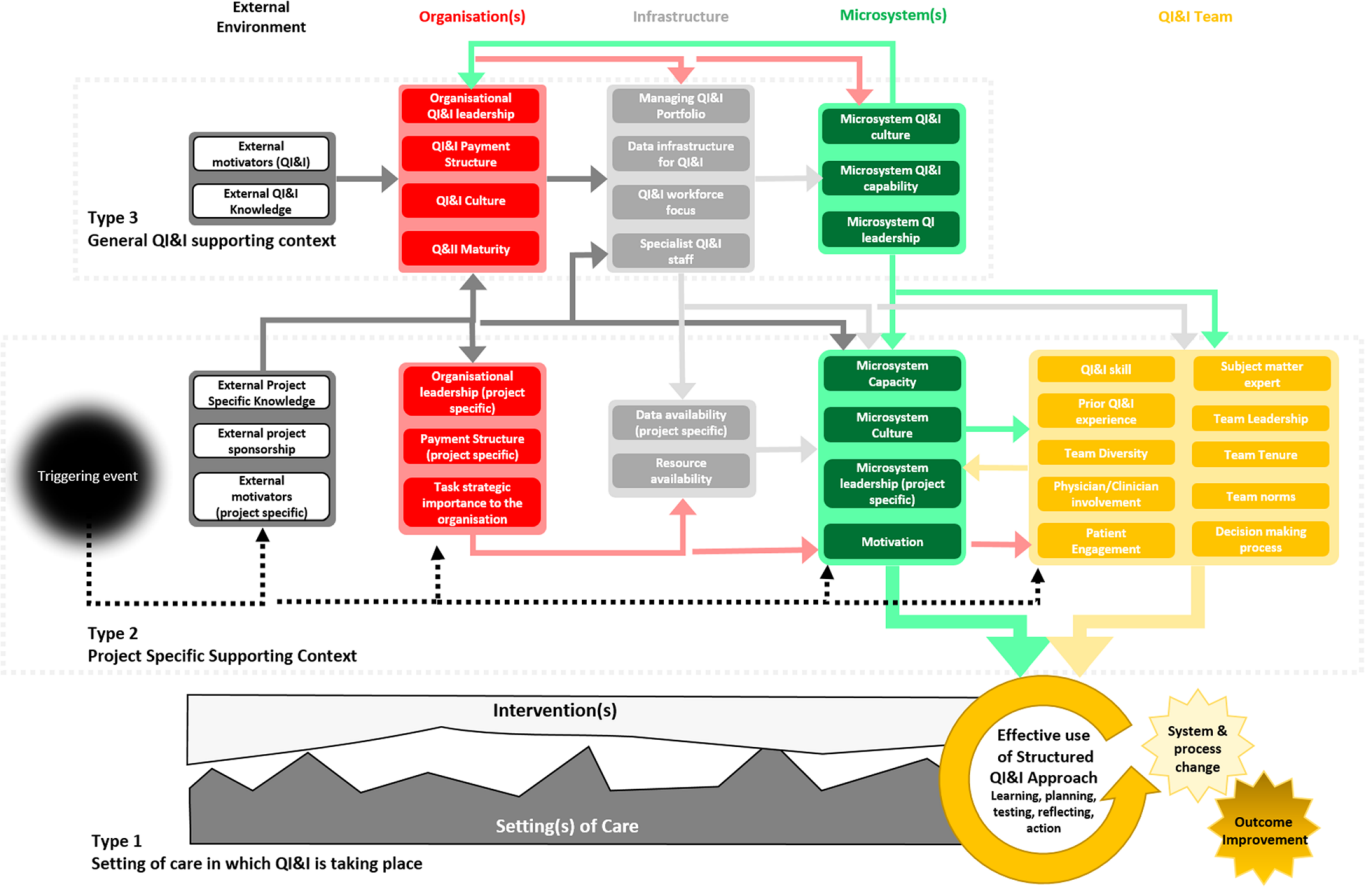
MUSIQv2.0 showing all factors aligned with Type 2 and Type 3 context, the relationship between factors, and their influence on the effective use of structured QI&I approaches to adapt interventions to local setting of care (Type 1 context) to achieve system and process change and outcome improvement

In its original form, MUSIQ focused mainly on context Types 2 and 3. Of the 36 updated MUSIQ factors we have classified 23 of the factors as Type 2 and 13 as Type 3. An updated model conceptualization of MUSIQ is provided in Fig. 2. The relationship between structured QI&I approaches and Type 1 context was not included in the original MUSIQ model. We noticed that we had less coding groups for Type 1 context and reflected that this maybe an artefact of our study designs which had sought to understand the influence of context on QI, rather than how QI&I approaches support the adaption of interventions to fit with local settings of care. Common Type 1 context themes emerged that related to modifying interventions to fit with practices or processes, making changes to related processes or policies, and modifying or adapting changes to take into account local capacity and capability. However, the specific requirements to modify an intervention or local care setting were observed to be different for each project or intervention. We therefore did not feel it was appropriate to identify common context factors for Type 1 context based on our analysis to date. We have instead represented this diagrammatically as the desire to fit interventions or changes to practice within the local setting.

## Discussion

In this qualitative exploration of MUSIQ, we confirmed that all original factors in MUSIQ were observed to be important in the QI initiatives studied and new factors were also identified. In addition, we identified three distinct types of context that are important in QI&I initiatives: the setting(s) of care in which QI&I takes place (Type 1); the context of the team conducting a specific project (Type 2); and the wider context supporting general QI&I (Type 3). The picture of context that emerges from the qualitative application of MUSIQ in real-world QI& I efforts is one that exhibits many of the classical features of a self-organising, complex system [39]. The context in which interventions are implemented has multiple elements, operating at different levels, and influencing improvement and implementation at different time points in ways that may bring about process and system-level change. Feedback and adaptation – particularly at the micro-system level – emerge as powerful influences on the direction of change for QI&I initiatives. Evaluating QI&I interventions with a complex system lens represents a significant step forward in addressing enduring controversies over the influence of context on QI&I initiatives and the approach to studying this phenomenon. A new version of MUSIQ (MUSIQ v2.0) was developed to better address this complexity.

### Context as a complex system

Importantly, the progress of improvement work in any given context – as outlined in Fig. 2 – is demonstrably non-linear, a key feature of a complex system. The relationships between the different types of context identified in this analysis are many and varied. The explicit articulation of distinct relationships between factors was a unique contribution of the previous model. Our qualitative analysis has extended the original model by revealing what these relationships look like, how different factors interact to influence project success, and importantly the ways in which these interactions may change over time. Each participant’s description of the role of context in their efforts revealed, either explicitly or implicitly, a dynamic situation in which factors are constantly interacting and feeding backwards or forwards across the types of context identified in Fig. 2, reinforcing the view of context as mediating and actively influencing improvement [14, 19]. Qualitative exploration allowed for a deeper understanding of these relationships that was not possible in previous quantitative studies and was masked by the simplification necessary when originally developing MUSIQ.

MUSIQv2.0 newly introduces the effective use of a structured QI&I approach as a focal point for understanding the influence of context and demonstrates how the structured QI&I approach is tightly linked to the three types of context identified in the study in different ways. The updated model provides a clear mechanism of action between contextual factors, the use of a structured approach, and the achievement of desired changes in system, processes and outcomes in a way that emphasizes the tight-linkages between these three aspects affecting change.

Because complex systems are often resistant to change on their own, any desired change must be deliberate and must be allowed to occur over time. Our studies revealed how attempts are made to intentionally modify aspects of context, reflecting greater or lesser degrees of resistance to change for each “type”. This is clearest in type 1 context where structured approaches are used to understand the setting of care and make modifications to the intervention or related care practices and process to support successful implementation. Our findings suggest that modifications to type 1 context can proceed comparatively rapidly over days, weeks or months. In type 2 context, teams can modify context by increasing levels of motivation or support for a specific project and organisation leaders can modify context in the way they support the team’s efforts. Effective use of structured QI&I approaches in and of itself can influence context at this level, for example by utilising data to create a tension for change and foster leadership support, or engaging staff in iterative development of solutions to increase their levels of motivation. These types of changes to type 2 context related to garnering support for a project or building relationships, typically occurs over months to years. Type 3 context is generally modified by organisational leaders who, for example, create an organisational direction for improvement and make investments in the strategic development of a culture of QI&I and supportive resources including specialist QI&I staff and data infrastructure. Modifications to type 3 context were noted to proceed slowly taking years and decades to influence and embed culture and infrastructure for improvement, which is consistent with previously studies of organisational development [17].

**Fig. 3:**
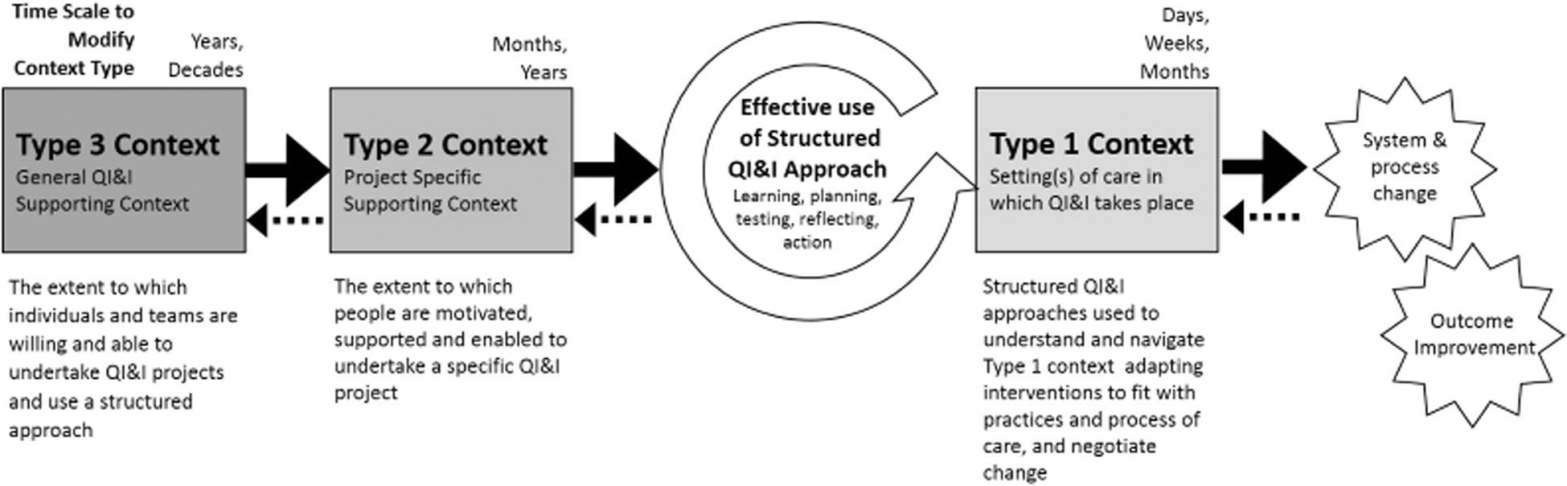
A schematic demonstrating the three types of context identified and how they are influenced by, or influence the effective use of QI&I approaches

Relationships – in the form of feedback and feedforward loops – between different context types were also observed. For example, strong leadership in relation to a specific project (Type 2) could have a positive impact on leadership in Type 3 context. The success or failure of a specific project to improve outcomes was also observed to impact motivation or resistance within Type 2 and 3 contexts. This demonstrates the relationships between context types and the complex and dynamic way in which they interact. The three types of context and their key features and relationship to QI&I approaches are presented in Fig. 3.

### Strengths and limitations of the analysis

The study is comparable in size to previous leading analyses of context in healthcare [8, 40–42] and was informed by a large and diverse sample of organisations and project types when compared to previous studies. Observations made in this study from both sources of qualitative data adds to the generalisability of the findings. Limitations of the study include the fact that both analysed studies were designed to view context through the lens of local QI projects. Although many projects in these studies were influenced, in part, by policy or financial (macro) drivers, further work to explore more policy driven initiatives may identify more environmental factors important for both project specific and general QI&I context. Secondly, the study utilised secondary analysis of qualitative data that had been collected primarily for other research studies. Whilst MUSIQ was used as a theoretical model to inform the design of both studies, there is a now a need to conduct primary research utilising this updated model (Fig. 2). Such research could also more explicitly target observations to understand further how context is intentionally modified, and to more directly explore how context impacts use of QI&I approach and project outcomes. Thirdly, while the data supports the importance of Type 1 context in QI&I success, we were unable to deeply explore themes underlying Type 1 context based on the type of secondary data available thus further research is required in this area. Finally, it was not possible to link findings from this analysis to project outcomes (for study B), so we cannot be definitive about which contextual factors are particularly prominent for interventions that are successful by comparison with those that are not.

MUSIQ v2.0 captures the intricate and complex relationship between context factors and how they influence QI&I efforts over time and provides insights to the practical reality of navigating and negotiating change. The three different types of context will have practical applications for those on the frontlines of QI&I by helping highlight the dynamic environment that teams are required to understand and navigate to support success of their projects. Further work is needed to understand how to translate and package these findings into useful tools to provide practical guidance to practitioners, managers and policy makers.

## Conclusion

In using MUSIQ to analyse qualitative data we found that context is complex, changing dynamically overtime, and is influenced by individual, team, organisation, and system characteristics and the relationships between them in a non-linear way – all distinctive features of a complex adaptive system. We were also able to identify three distinct types of context including, the setting(s) of care in which QI&I take place (Type 1), the context of the team conducting a specific project (Type 2); and the wider context supporting general QI&I (Type 3). These insights have resulted in a new version of the model, MUSIQ v2.0, which may help address some existing controversies about how context influences QI&I success and help ensure that future research efforts consider context not as a static background factor, but as a complex system that is constantly changing, tightly linked, and governed by feedback loops.

## Additional file

Additional file 1: Definition of revised MUSIQ Type 2 (project specific) and Type 3 (general QI&I) context factors. Definitions of each factor and classification compared to original MUISQ framework factors (same, modified, new). (DOCX 16 kb)

## Abbreviations

MUSIQ: Model for Understanding Success in Quality
NHS: National Health Service
OPQC: Ohio Perinatal Quality Collaborative
PDSA: Plan-do-study-act
QI: Quality Improvement
QI&I: Quality Improvement and Implementation
