# Supplementary material for: A new typology for understanding context: qualitative exploration of the model for understanding success in quality (MUSIQ)
Source: BMC Health Serv Res. 2018 Jul 25;18:584. doi: 10.1186/s12913-018-3348-7 (PMC6060552; doi:10.1186/s12913-018-3348-7)
Supplement: Supplementary file 1 — Definition of revised MUSIQ Type 2 (project specific) and Type 3 (general QI&I) context factors. Definitions of each factor and classification compared to original MUISQ framework factors (same, modified, new). (DOCX 16 kb) [file 12913_2018_3348_MOESM1_ESM.docx]

**Additional File 1**

Definition of revised MUSIQ Type 2 (project specific) and Type 3 (general QI&I) context factors. Key S = the factors is the same as the original; M = the factor has been modified from the original; N = the factor is new.

| **Type 2 Factors** | | | **Type 3 Factors** | | |
| --- | --- | --- | --- | --- | --- |
| External Environment | | | | | |
| External Motivators (project specific) | S | Environmental pressures and incentives that stimulate the organisation to improve its performance and quality in the area of focus of this QI&I project | External Motivators (QI&I) | N | Environmental pressures and incentives that stimulate the organisation to improve its support for QI&I |
| External Knowledge (project specific) | N | The extent to which the team or organisation values and acquires project related knowledge from external sources (e.g. guidelines, other project teams, other hospitals) | External Knowledge (QI&I) | N | The extent to which the team or organisation values and acquires quality improvement knowledge from external sources (e.g. publications, other QI&I organisations) |
| External Project Sponsorship | S | Substantial and meaningful contributions of personnel, expertise, money, equipment, facilities, or other important resources from outside entities (external to the organisation) with formal relationships with this QI&I project |  |  |  |
| Organisation | | | | | |
| Organisational Leadership (project specific) | M | Senior management's (CEO, COO, CMO, Senior VP) oversight, direction setting, and support in removing barriers and overcoming obstacles of specific QI&I efforts. | Organisational QI&I Leadership | S | Senior management’s (CEO, COO, CMO, Senior VP, Board of Directors) governance, guidance, support, oversight, and direction setting of all QI&I efforts |
| Task Strategic Importance to Organisation | S | Work perceived as part of the organisation’s strategic goals | Organisational QI&I Maturity | S | Sophistication of the organisation’s QI&I programme including organisational memory |
| Payment Structure (project specific) | M | Physicians and other clinicians are employed by and/or receive monetary compensation from the organisation in a way that supports the specific project | Payment Structure (QI&I) | M | Physicians and other clinicians are employed by and/or receive monetary compensation from the organisation in a way that supports the engagement in QI&I |
|  |  |  | Organisational QI&I Culture | S | Values, beliefs, and norms of an organisation that shape the behaviours of staff in pursuing QI&I I |
| Infrastructure | | | | | |
| Data available to guide project | N | Extent to which data is available and accessible to support a specific improvement project | Data Infrastructure for QI&I | S | Extent to which systems exists to collect, manage, and facilitate the use of data needed to support performance improvement |
| Resource availability (project specific) | S | Degree to which financial support for QI&I, including allocation of resources and staff time, is provided for a specific project | Specialist QI&I staff | N | The availability, expertise and experience of staff with specialist or high levels of quality improvement and implementation knowledge and skills |
|  |  |  | QI&I Workforce Focus | S | Degree to which the organisation develops the workforce through training and engages them in QI&I through reward systems and expectation setting |
|  |  |  | Managing QI&I Portfolio | N | The extent to which an organisation has a system for managing multiple QI&I projects including processes for selecting projects and appropriate quality improvement methods to use |
| Microsystem | | | | | |
| Microsystem Leadership (project specific) | N | Degree to which microsystem leadership are personally involved in supporting and facilitating a specific QI&I effort | Microsystem QI&I Leadership | S | Microsystem leadership capacity for QI&I and degree to which they are personally involved in supporting and facilitating all QI&I efforts |
| Microsystem Capacity (project specific) | N | Microsystem staff's collective potential for delivering care and executing this specific project | Microsystem QI&I Capability | S | Microsystem staff’s ability to use QI&I methods for change |
| Microsystem Culture | N | Values, beliefs, and norms present in the microsystem that emphasise patient centeredness, cooperative relationships among staff in the microsystem, and collective responsibility for patient care. | Microsystem QI&I Culture | S | Values, beliefs, and norms present in the microsystem that emphasise teamwork, communication, freedom to make decisions, and commitment to improve |
| QI&I Team | | |  | | |
| Prior QI&I Experience | S | Prior experience with QI&I |  |  |  |
| Team Diversity | S | Diversity of team members with respect to professional discipline, personality, motivation, and perspective |  |  |  |
| Physician and Clinician Involvement | M | Contribution of physicians and other healthcare professionals to the QI&I team efforts |  |  |  |
| Patient Engagement and Involvement | N | Contribution of patients, carers and members of the public to the QI&I team efforts |  |  |  |
| Team Leadership | S | Team leader’s ability to accomplish the goals of the improvement project through guiding the actions of the QI&I team |  |  |  |
| Decision Making Processes | S | Team engages in well-designed decision-making practices |  |  |  |
| Team Norms | S | Team establishes strong norms of behaviour related to how work is to be carried out and how goals are to be achieved |  |  |  |
| QI&I Skill | S | Team’s ability to use improvement and implementation methods to make changes |  |  |  |
| Team Tenure | S | Team members have worked together as a team before |  |  |  |
| Subject Matter Expert | S | One or more team members is knowledgeable about the outcome, process, or system being changed |  |  |  |
